# Mechanotransduction and musculoskeletal regeneration: Molecular mechanisms and interdisciplinary applications

**DOI:** 10.1016/j.gendis.2025.101731

**Published:** 2025-06-22

**Authors:** Xiajie Huang, Wenjun Hao, Yangzhou Mo, Xinyun Liang, Xiaomei Wu, Daofu Zeng, Yubin Mo, William Lu, Di Chen, Yan Chen

**Affiliations:** a Department of Bone and Joint Surgery, The First Affiliated Hospital of Guangxi Medical University, Nanning, Guangxi 530021, China; b Collaborative Innovation Centre of Regenerative Medicine and Medical BioResource Development and Application Co-constructed by the Province and Ministry, Guangxi Medical University, Nanning, Guangxi 530021, China; c Department of Orthopaedics and Traumatology, The University of Hong Kong, Hong Kong 999077, China; d Research Center for Computer-aided Drug Discovery, Shenzhen Institute of Advanced Technology, Chinese Academy of Sciences, Shenzhen, Guangdong 518055, China

**Keywords:** Cross-organ regeneration, Distraction histogenesis, Distraction osteogenesis, Interdisciplinary applications, Law of tension-stress, Mechanosensitive channels, Mechanotransduction

## Abstract

Distraction osteogenesis, or the Illizarov technique, induces bone regeneration using distractive mechanical forces. Nevertheless, Wolff's law holds that bone adapts to reverse compressive mechanical loads, growing denser in areas of high pressure and resorbing in zones of low pressure. These two forms of new bone formation together suggest that mechanical stimuli play an important role in bone remodeling and regeneration. The therapeutic efficacy of distraction osteogenesis has been recognized in orthopedics and maxillofacial surgeries. Distraction osteogenesis was even used for the regeneration of various other tissues/organs, such as blood vessels and skin (*e.g.*, in the treatment of limb ischemic diseases and foot ulcers), suggesting the principle of distraction histogenesis. However, the underlying mechanisms, particularly those of the cross-organ effects and in terms of mechanotransduction, remain poorly understood. Thus, this review aims to explore the recent advances in research on musculoskeletal regeneration and its association with mechanosensitive channels from a new interdisciplinary application perspective. The contents can provide insights into potential research directions for understanding the molecular mechanisms of musculoskeletal regeneration and its clinical applications.

## Introduction

Distraction osteogenesis (DO), which was introduced by Dr. Ilizarov in the mid-20th century and also known as bone transport, the law of tension-stress, or Ilizarov technique, induces bone regeneration via an osteotomy followed by distracted mechanical forces.^1^^,^^2^ Interestingly, Wolff's law, developed by Julius Wolff in the 19th century, states that bone remodels itself to the mechanical loads (*i.e.*, the reverse compressive forces), growing denser in areas of high pressure and resorbing in areas of low pressure.^3^^,^^4^ Nevertheless, no matter whether the new bone is mediated by distraction according to the DO principle or by compression based on Wolff's law, there is a consensus that mechanical stimuli play an important role in bone remodeling and regeneration.^5^

DO has been successfully used in many fields such as bone defect repair, deformity correction, limb lengthening, and orthodontics.^6^, ^7^, ^8^, ^9^, ^10^ Moreover, the principles and applications of DO have extended to the regeneration of blood vessels, skin, nerves, and other tissues.^11^ This leads to the purpose of the distraction histogenesis (DH) theory, which holds that the distraction of any living tissues leads to the regeneration of the tissues themselves. Furthermore, recently, we and other groups have found that tibial cortex transverse transport (TTT) facilitates the healing of recalcitrant ulcers at remote sites, such as the foot.^12^, ^13^, ^14^, ^15^, ^16^ These interdisciplinary and cross-organ effects underscore the universal importance of mechanical stimulation in tissue regeneration. During DH, bones serve as sites for mechanical force stimulation and formation of multiple osteokines, showing the central role of mechanical signals in promoting regeneration and repair.^17^^,^^18^ To promote tissue regeneration, mechanical signals need to be converted into biological signals by mechanosensitive channels, a process known as mechanotransduction.^19^ Understanding the mechanism of mechanotransduction in DH is crucial for advancing its application.^18^^,^^20^ Despite the widespread clinical application of DH, the mechanistic pathways, particularly those involving mechanosensitive channels, are not well documented in the existing literature. Hence, this review explores the recent advances in research on DH and its relationship with mechanosensitive channels, highlighting their roles in mechanotransduction.

## Research progress in distraction histogenesis

### Development of DH

**Figure 1:**
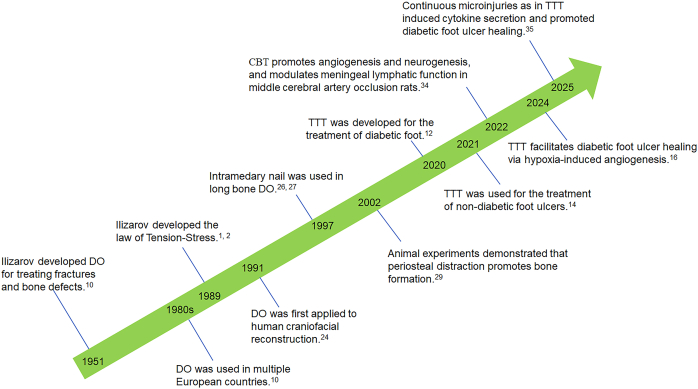
The development of DO and DH. DO, distraction osteogenesis; DH, distraction histogenesis; TTT, tibial cortex transverse transport; CBT, cranial bone transport.

DO was initially used for the treatment of complex fractures and bone defects^10^^,^^21^ (Fig. 1). This technique gained international recognition in the 1980s and began to be used for a wide range of clinical settings, including bone defect repair, limb lengthening, and various bone deformity corrections.^10^^,^^22^^,^^23^ In 1989, Dr. Ilizarov elaborated the law of tension-stress, which states that applying continuous, stable, and slow distraction forces to living tissues can stimulate their own regeneration and growth.^1^^,^^2^ DO was then used for human mandibular elongation in 1991.^24^^,^^25^ Subsequently, DO has been widely used for the reconstruction of craniofacial bone defects and deformities, limb deformity correction, and lengthening^26^, ^27^, ^28^, ^29^, ^30^, ^31^ (Fig. 1).

**Figure 2:**
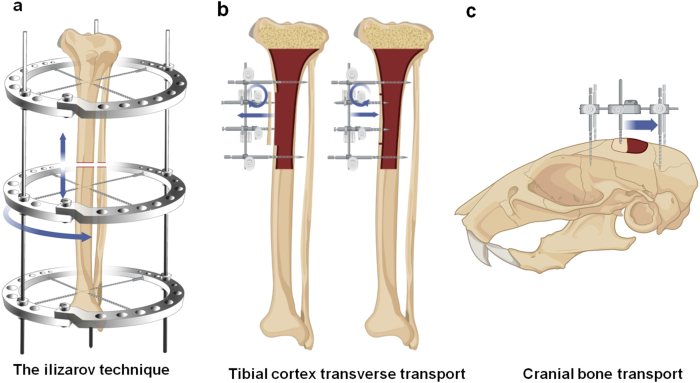
Comparative models of Ilizarov technique, tibial cortex transverse transport, and cranial bone transport. **(A)** The traditional Ilizarov technique employs circular external fixators to gradually apply longitudinal tension to osteotomized bone segments, promoting regeneration of bone itself and the surrounding soft tissues. **(B)** TTT technique utilizes unilateral external fixators to slowly laterally transport tibial bone segments, fostering regeneration of vascular tissues and skin at the foot wounds. **(C)** Cranial bone transport enhanced angiogenesis, neurogenesis, and meningeal lymphatic drainage in rats with cerebral ischemia, suggesting it is a potential therapeutic approach for cerebral ischemic diseases.

Recently, Chinese scholars developed the TTT.^12^, ^13^, ^14^, ^15^, ^16^^,^^32^ Unlike the traditional longitudinal bone transport, in TTT, the bone fragments were distracted horizontally/transversely (Fig. 2A, B). Furthermore, the bone traction frame used in TTT is unilateral, which simplifies the procedure compared with the more complex circular frame used in the Ilizarov technique. This simplicity benefits both the surgical process and postoperative management, making it more accessible and acceptable to patients. The primary aim of TTT is not to stimulate bone formation, but rather, the regeneration of the soft tissues or organs, including skin, vessels, nerves, and muscle.^15^ This technique is particularly effective in treating chronic ischemic diseases of the lower extremities, such as diabetic foot ulcers, thromboangiitis obliterans, and arteriosclerotic obliterans.^32^^,^^33^ This is a key focus of our group's research and efforts.^12^, ^13^, ^14^, ^15^, ^16^ In addition, cranial bone transport was found to enhance angiogenesis, neurogenesis, and meningeal lymphatic drainage in rats with cerebral ischemia^34^ (Fig. 2C), suggesting that DO could serve as a novel therapeutic approach for cerebral ischemic diseases. Together, the prior findings indicate that the distraction of any living tissues leads to the regeneration of the tissues themselves. Thus, the term distraction histogenesis (DH) is coined for this theory.

DO consists of continuous microinjuries (microinjury-new bone formation cycles).^35^ Injury can trigger *in situ* tissue repair, leading to the restoration of tissue structure and functions at the injury site through a series of well-orchestrated biological events.^36^ More intriguing is the concept that microinjury can mediate localized tissue regeneration without resulting in severe trauma or scarring.^37^ This concept is supported by evidence that microinjuries can stimulate the body's natural repair mechanisms, activating cellular responses and tissue growth in a localized manner, similar to larger injuries, but with less risk of fibrosis or adverse side effects.^36^^,^^37^

Based on the findings of prior studies, we propose a theory of microinjury-induced remote repair.^35^ This theory holds that remote continuous microinjuries performed intentionally in normal tissues (*e.g.*, bone) can trigger the intrinsic repair/regeneration ability of the body, which facilitates the repair/regeneration of not only the *in situ* tissues (*e.g.*, bone and the surrounding soft tissues) but also the target tissues (*e.g.*, foot ulcers).^35^ The microinjuries created should follow some instructions as summarized in Table 1. Nevertheless, this theory needs to be validated, and our studies are ongoing.

**Table 1:** Key characteristics of the microinjury-induced remote repair (MIRR) theory.

Characteristics	Description
Microinjuries	Injuries should be small enough to trigger pro-regenerative cytokine production while not causing severe local damage or scar formation.
Continuous injuries Controlled injuries	Injuries must be persistent to sustain cytokine release, aligning with the prolonged healing process of target tissues. Injuries should be controllable by doctors and/or patients or self-administrable (*e.g.*, using external distraction system).
Remote location	Injuries should be distant from the target damaged tissues to avoid further trauma and minimize infection risk of the surgical site.
Injury purpose	Mimics the body's intrinsic repair mechanisms to enhance tissue regeneration at both local and distant sites.

### Traditional applications of DH

#### Bone defect repair

**Figure 3:**
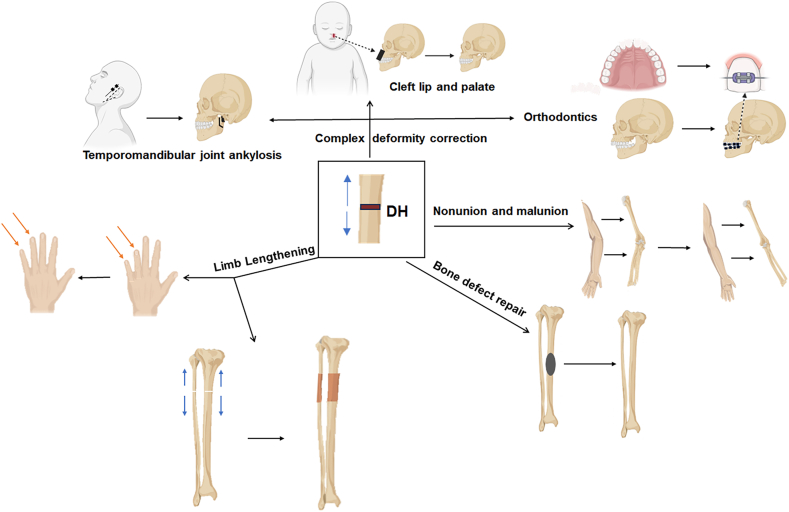
The traditional applications of distraction histogenesis.

DH is extensively used for bone defect repair, particularly in craniofacial and orthopedic surgeries (Fig. 3). This technique is valuable in cases of severe bone loss caused by trauma, infections, or tumor resection, as it can regenerate bone at major defect sites without extensive bone grafts.^38^, ^39^, ^40^

#### Limb lengthening

Limb lengthening surgery involves gradual distraction of the osteotomized bone segments, promoting abundant new bone formation at the gap, thus achieving an increase in bone length.^41^^,^^42^ This technique has been successfully used for various congenital and acquired conditions affecting the clavicle, humerus, radius, ulna, and phalanges (Fig. 3). Common indications for limb lengthening include chondrodysplasia, radial longitudinal deficiency, multiple hereditary exostoses, short metacarpal bones, and symbrachydactyly, as well as post-traumatic and post-infection growth disturbances.^43^^,^^44^

#### Deformity correction

DH effectively corrects complex bone deformities and can be applied to various anatomical locations, including craniofacial areas, the spine, and long bones^45^^,^^46^ (Fig. 3). This technique allows precise control over the extent and direction of bone growth. In craniofacial reconstruction, DH is used for patients with conditions such as cleft lip and palate, improving their appearance and functionality.^47^, ^48^, ^49^ In orthognathic surgery, this technique is used for mandibular advancement procedures to improve occlusion and facial aesthetics, particularly useful for treating temporomandibular joint disorders and jaw asymmetry.^25^ Additionally, DH can be utilized in orthodontic treatments to adjust the shape and position of the alveolar bone to correct tooth alignment.^50^^,^^51^

#### Treatment of nonunion and malunion

In the treatment of nonunion and malunion, DH offers significant advantages (Fig. 3). DH effectively stimulates bone generation through sustained mechanical traction and improves the blood supply and metabolism of bone tissue, thereby accelerating the healing process. Compared with traditional methods such as bone grafting and internal fixation, DH not only reduces the risks of rejection and infections but also significantly increases the bone healing rate by utilizing the patient's own ability for regeneration.^52^, ^53^, ^54^, ^55^

### The recent applications of DH

#### Management of ischemic diseases

**Figure 4:**
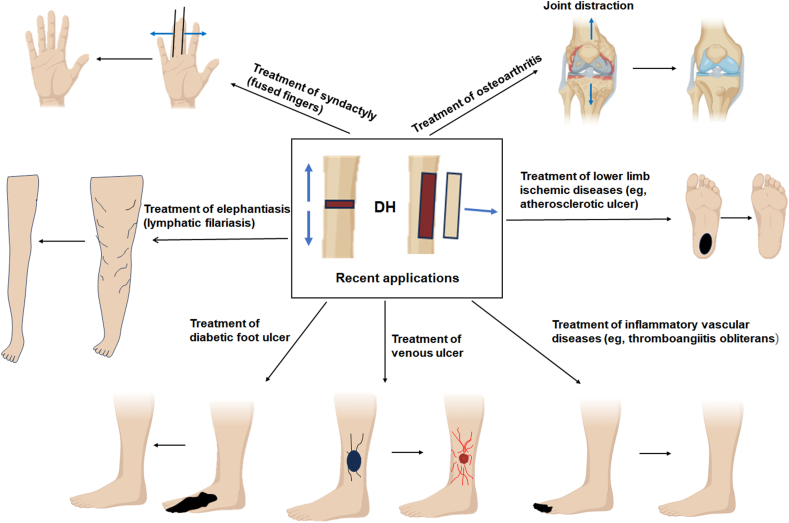
The recent applications of distraction histogenesis to soft tissue regeneration.

DH can be used to improve ischemic diseases of the lower limbs by gradually lengthening the bone around the ischemic area, thereby increasing vascular generation and blood supply and improving tissue repair and symptom relief^56^^,^^57^ (Fig. 4). For severe arteriosclerosis obliterans of the lower limbs, traditional treatments primarily involve interventional surgery to reopen the large blood vessels near the proximal limb. However, it is technically challenging to reopen smaller blood vessels in the distal limb (*e.g.*, below the ankle), and the treatment results are frequently unsatisfactory. DH can promote microvascular regeneration, achieving significant therapeutic effects in the treatment of severe arteriosclerosis obliterans of the lower limbs.^56^^,^^57^

#### Treatment of refractory wounds

Refractory wounds are traumatic wounds that cannot heal or cure on their own.^58^^,^^59^ They can be caused by various factors such as infection, ischemia, or diabetes.^60^ DH can accelerate the healing process of such refractory wounds by altering the structure of surrounding tissues and promoting vascular generation (Fig. 4). In some cases, DH can provide sufficient regenerated tissues to ultimately facilitate wound healing, thereby avoiding complications such as infection or necrosis.^12^, ^13^, ^14^, ^15^^,^^61^

In addition, studies have shown that TTT has demonstrated good efficacy in the treatment of lower limb lymphedema^62^ (Fig. 4). Other reports indicate that with the assistance of a simple Ilizarov external fixator, sufficient skin can be gradually stretched to reconstruct web spaces and cover lateral defects of the fingers^63^ (Fig. 4). Through structured functional rehabilitation, patients not only regained hand function but also achieved satisfactory aesthetic outcomes.^63^ Remarkably, the application of DH technology has also been extended to the management of osteoarthritis. Joint distraction surgery, which uses an external fixator to maintain joint space and reduce cartilage loading, has shown promising long-term results in patients with end-stage osteoarthritis (Fig. 4). Compared with total knee arthroplasty, knee joint distraction has provided a satisfactory joint-preserving alternative based on long-term follow-up studies.^64^^,^^65^ Overall, DH is a versatile medical technique that has been widely applied across various fields and continues to drive advancements in the medical sector.

### Cellular responses during DH

During DH, mechanical distraction stimuli activate various cell types to participate in the repair and regeneration of both bone and soft tissues (including vessels, skin, and nerves). The following summarizes the specific responses and mechanisms of different cellular populations involved in this process (Table 2).

**Table 2:** Cellular responses during distraction histogenesis.

Cell type	Cellular responses and mechanisms during distraction histogenesis	References
Bone marrow mesenchymal stem cells	Respond to mechanical tension with chemotaxis, leading to proliferation and differentiation toward osteogenic and angiogenic lineages; secrete exosomes to support angiogenesis and osteogenesis.	66, 67, 68, 69, 70
Osteoblasts	Directly participate in new bone formation; mechanical tension enhances their proliferation, matrix secretion, and mineralization.	70, 71, 72
Osteocytes	Act as primary mechanosensors embedded in bone matrix; detect mechanical stretch via Piezo1, triggering downstream osteogenic signals.	68
Chondrocytes	Involved in both intramembranous and endochondral ossification; promote cartilaginous callus formation and extension via chondromodulin expression.	73,74
Endothelial cells and endothelial progenitor cells	Undergo proliferation, migration, and lumen formation in response to mechanical stimulation; endothelial progenitor cells mobilize to injury sites to promote angiogenesis.	16,35,75, 76, 77
Fibroblasts	Proliferate and synthesize collagen and fibronectin; contribute to extracellular matrix remodeling and provide structural support and mechanical cushioning.	61,78,79
Myogenic cells	Activated by mechanical stretch; promote muscle tissue repair, muscle fiber formation, and regeneration.	80
Macrophages and immune cells	Mechanical forces promote M2 polarization; M2 macrophages suppress excessive inflammation, enhance angiogenesis and osteogenesis; mechanical stimuli activate immune cells to modulate the microenvironment.	70,77,81, 82, 83
Neural cells (Schwann cells)	Activated by distraction forces; secrete neurotrophic factors that promote axonal regeneration and myelin repair.	84, 85, 86, 87

#### Bone marrow mesenchymal stem cells

Bone marrow mesenchymal stem cells (BMSCs) are one of the key mechanosensitive multipotent stem cell populations and serve as the critical starting point for distraction osteogenesis and multi-tissue regeneration.^66^^,^^67^ Mechanical tension stimulates the chemotaxis, proliferation, and differentiation of BMSCs toward osteogenic and angiogenic lineages.^66^^,^^67^ This differentiation process is primarily mediated through the activation of the Wnt/β-catenin and transforming growth factor-beta (TGF-β)/bone morphogenetic protein (BMP) signaling pathways.^66^^,^^67^ In addition, the piezoelectric element-type mechanosensitive ion channel Piezo1 plays an essential role in enabling BMSCs to sense mechanical stretch; its activation enhances the osteogenic potential of BMSCs and promotes new bone formation.^68^ Recent research also suggests that BMSCs contribute to angiogenesis and osteogenesis by secreting exosomes, playing a vital role throughout the entire process of distraction-induced tissue regeneration.^69^^,^^70^

#### Osteoblasts, osteocytes, and chondrocytes

During DH, osteoblasts serve as key effector cells directly responsible for new bone formation.^70^, ^71^, ^72^ Mechanical tensile forces significantly enhance their proliferation, bone matrix secretion, and mineralization.^70^, ^71^, ^72^ Simultaneously, osteocytes, embedded within the bone matrix as primary mechanosensors, can detect tensile forces via the activation of Piezo1 ion channels.^68^ This mechanotransduction process further stimulates the osteogenic differentiation of BMSCs, thereby promoting bone regeneration.^68^ In parallel, chondrocytes also play an essential role during DH. They are involved in both intramembranous and endochondral ossification and further contribute to tissue regeneration by up-regulating chondromodulin expression, which promotes the formation and extension of cartilaginous callus.^73^^,^^74^

#### Endothelial cells and endothelial progenitor cells

Angiogenesis is an essential process during DH. Studies showed that mechanical stimulation can activate the vascular endothelial growth factor (VEGF), hypoxia inducible factor-1alpha (HIF-1α), and phosphatidylinositol 3' -kinase (PI3K)/protein kinase B (Akt) signaling pathways, inducing endothelial cell proliferation, migration, and lumen formation.^16^^,^^75^^,^^76^ In addition, during transverse bone transport in the rat tibia, bone marrow-derived endothelial progenitor cells can be activated and proliferate, subsequently mobilizing into the peripheral circulation to reach diabetic foot ulcers, where they promote vascular regeneration.^35^^,^^77^

#### Fibroblasts and myogenic cells

During DH, in addition to the regeneration of bone and vessels, the repair of soft tissues such as skin and muscle is equally critical. Fibroblasts proliferate under mechanical distraction stimuli and synthesize collagen and fibronectin, contributing to extracellular matrix remodeling and providing structural support and mechanical buffering for newly formed tissues.^61^^,^^78^^,^^79^ Meanwhile, myogenic cells are also activated by mechanical stretch, promoting muscle tissue repair and facilitating the formation and regeneration of muscle fibers.^80^

#### Macrophages and immune cells

Mechanical forces play a crucial role in tissue repair and regeneration by regulating macrophage polarization in DH. These forces promote the polarization of macrophages from M1 toward the M2 phenotype, which helps suppress excessive inflammatory responses while enhancing angiogenesis and osteogenesis.^77^^,^^81^ In addition, micro-injuries and continuous mechanical stimulation during the distraction process synergistically activate immune cells, modulate the local microenvironment, and thereby facilitate tissue remodeling and regeneration^70^^,^^82^^,^^83^

#### Neural cells

In recent years, studies have found that mechanical distraction can also influence the regeneration of the peripheral nervous system.^84^, ^85^, ^86^, ^87^ Schwann cells are activated under distraction conditions and secrete neurotrophic factors, such as nerve growth factor (NGF) and brain-derived neurotrophic factor (BDNF), which promote axonal growth and myelin repair.^84^, ^85^, ^86^, ^87^

## Mechanosensitive channels

Mechanosensitive channels are a specialized class of protein channels that sense physical forces such as pressure, stretch, or tactile stimuli and convert them into intracellular signals, which are crucial for bone and tissue regeneration.^88^, ^89^, ^90^ These channels are embedded in the cell membrane and open in response to physical deformation caused by external forces, allowing ions like sodium and calcium to flow in or out of the cell, thereby initiating intracellular signal transduction and other physiological responses.^88^, ^89^, ^90^ These channels are widespread among bacteria, archaea, and eukaryotes, playing a significant role in evolutionary history and signal transduction.^91^ The mechanosensitive channels have four widely accepted standards: i) the channels must be expressed in mechanosensory organs; ii) removing mechanosensitive channels results in the direct loss of mechanical responses; iii) mutation of these channels alters their physical properties and reflects in mechanical response changes; and iv) heterologous expression of channels can be mechanically activated.^92^

Historically, most knowledge about the gating mechanisms of mechanosensitive channels came from studies on bacterial channels. However, recent advances in identifying and understanding the structure of eukaryotic two-pore domain K^+^ (K2P) channels, Twik-related K^+^ (TREK) and Twik-related acid-arachidonic activated K^+^ (TRAAK), as well as Piezo1 and Piezo2 mechanosensitive channels, have greatly enhanced our understanding of the biophysical principles of these fascinating membrane proteins and their evolutionary origins.^91^ To date, several mechanogated channel families, such as degenerin/epithelial sodium (DEG/ENaC), transient receptor potential (TRP), K2P, transmembrane channel-like (TMC), and Piezo channels, have been identified in eukaryotes.^92^ The following sections will mainly introduce three mechanosensitive channels closely related to DH: Piezo channels, TRP channels, and ENaCs.^93^ We summarize the key mechanosensitive channels and their functions in DH in Table 3.

**Table 3:** Key mechanosensitive channels and their functions in distraction histogenesis.

Mechanosensitive channels	Key channel	Stimulus	Ions involved	Functions	Location	Discovery year	References
Piezo	Piezo1	Mechanical force, physical stimuli	K^+^, Na^+^, Ca^2+^, Mg^2+^	Activating downstream osteogenic pathways	Osteoblasts, bone marrow mesenchymal stem cells, osteocytes, chondrocytes, periodontal ligament cells, and periodontal ligament fibroblasts	2010	20,98, 99, 100,121,132
TRP	TRPV4, TRPM7	Mechanical stimulation	Ca^2+^	Activating downstream osteogenic pathways	Osteocytes, osteoblasts, osteoclasts, and chondrocytes	1997	136,139, 140, 141,143,135,155, 156, 157
ENaC	/	Shear force, PH	Na^+^	Still unclear	Osteocytes, osteoblasts, osteoclasts, and chondrocytes	1993	93,160, 161, 162,166

Note: Piezo, piezoelectric element; ENaC, epithelial sodium; TRP, transient receptor potential.

### Piezo channels

**Figure 5:**
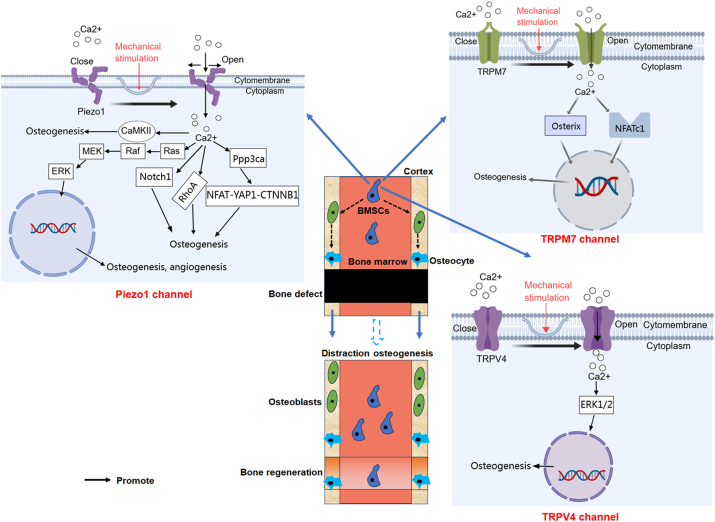
During the distraction histogenesis process, the Piezo1, TRPM7, and TRPV4 channels are activated in BMSCs and trigger downstream osteogenic pathways. Piezo, piezoelectric element; TRP, transient receptor potential; BMSCs, bone marrow mesenchymal stem cells.

Identified firstly in 2010, the Piezo channel family primarily consists of Piezo1 and Piezo2, both of which play a crucial role in mechanotransduction.^20^^,^^94^ In humans, Piezo1 and Piezo2 consist of 2521 and 2752 amino acids, respectively, while in mice, these channels have 2547 and 2822 amino acids.^95^^,^^96^ Piezo channels are highly sensitive to mechanical stimuli and are extensively distributed among mammalian cells.^20^^,^^97^ Upon activation by mechanical stimuli, Piezo ion channels allow the influx of various ions such as K^+^, Na^+^, Ca^2+^, and Mg^2+^ into the cell^97^, ^98^, ^99^, ^100^, ^101^ (Fig. 5). This influx occurs because the Piezo1 channel forms a pore that opens in response to mechanical forces, permitting these extracellular ions to enter the cell and thereby initiating various downstream signaling pathways.^98^, ^99^, ^100^ Despite sharing a similar trimeric structure, there are differences between Piezo1 and Piezo2, as revealed by cryo-electron microscopy studies on mouse Piezo1 and Piezo2.^95^^,^^96^^,^^102^^,^^103^ Consequently, their functions and roles also vary. In lung expansion and blood flow, Piezo1 plays a more critical role than Piezo2.^103^, ^104^, ^105^, ^106^, ^107^ Studies on mice with Piezo1 and Piezo2 knockouts found Piezo1 to be a key channel in responding to mechanical stimuli to enhance osteogenesis, in which mechanical forces triggered Piezo1 to enhance family with sequence similarity 20 member C (FAM20C) kinase production and dentin matrix protein 1 (DMP1) secretion.^108^ Experiments under simulated microgravity conditions further confirmed the importance of Piezo1 in maintaining osteoblastic cell function.^109^ Notably, patients with osteoporosis exhibit reduced Piezo1 expression, suggesting its necessity in normal osteoblastic cell functions.^109^ Studies have also found that activation of the Piezo1-Akt pathway plays a critical role in the mechanical stretch-induced down-regulation of sclerostin (Sost) expression, thereby reducing osteocyte apoptosis.^110^^,^^111^ Another study on mouse models with endothelial-specific loss of Piezo1 showed that its absence not only affected fracture repair but also inhibited the maturation process of osteoblastic cells, suggesting Piezo1 as a potential target for promoting bone regeneration and fracture healing.^112^ In addition, Piezo1-mediated mechanotransduction activates calcium/calmodulin-dependent protein kinase II (CaMKII) signaling in ankylosing spondylitis patients, promoting pathological new bone formation at the attachment points.^113^ Recent studies further indicate that Piezo1 can promote osteogenesis by activating the CaMKII signaling pathway in response to mechanical stimulation.^109^^,^^113^, ^114^, ^115^, ^116^ These findings collectively highlight Piezo1 as a critical mechanical sensor.

Piezo1 is expressed in various cell types within bone tissue.^117^ In osteoblasts, Piezo1 plays a critical role, particularly in regulating bone formation under mechanical stimulation.^118^ Osteoblasts are the primary cells responsible for bone generation, and when Piezo1 senses mechanical stress, it activates downstream signaling pathways, promoting the deposition and mineralization of the bone matrix.^109^^,^^119^^,^^120^ Piezo1 is also expressed in BMSCs and may promote osteogenic differentiation through the mitogen-activated protein kinase (MAPK) pathway.^121^ Studies have shown that Piezo1 promotes the differentiation of BMSCs into osteoblasts in response to mechanical stimulation while inhibiting adipocyte differentiation.^119^^,^^122^^,^^123^ Research indicates that in the context of BMSCs, under mechanical stimulation, Piezo1/2 regulates the coordinated activation of nuclear factor of activated T cells 1 (NFATc1), Yes-associated protein-1 (YAP1), and catenin beta 1 (CTNNB1) via protein phosphatase 3 catalytic subunit alpha (Ppp3ca), promoting osteoblast differentiation.^124^ Additionally, in mesenchymal stem cells (MSCs), Piezo1 further enhances osteogenesis by activating the Ras homolog family member A (RhoA) pathway in response to mechanical forces.^125^ Additionally, in periodontal ligament stem cells, mechanical force activates Piezo1 protein, which mediates osteogenic differentiation through the Notch signaling pathway.^126^ In chondrocytes, Piezo1 is involved in mechanosensing and the regulation of cellular metabolism, particularly in the growth plate region and cartilage tissue.^127^ It plays an important role in bone development and the mechanical properties of the skeleton.^127^ Piezo1 also has a crucial function in osteocytes, which are located within the bone matrix and are continuously exposed to mechanical stress. Through Piezo1, osteocytes can sense and respond to mechanical forces, thereby regulating bone remodeling. Overall, Piezo1 plays a vital role in mechanosensing across different cell types in bone tissue, helping to regulate bone formation and remodeling processes.

In the field of orthodontics, the key role of the Piezo1 channel in orthodontic tooth movement has been identified.^128^ Activation of this channel not only promotes new alveolar bone formation during orthodontic procedures but also plays a key role in maintaining tooth movement speed.^129^ Additionally, the study found that Piezo1 may be involved in the mechanotransduction of periodontal ligament cells through the MAPK signaling pathway.^130^^,^^131^ When periodontal ligament fibroblasts are subjected to mechanical stimulation, Piezo1 can also regulate osteoclastogenesis.^132^ These discoveries underscore the importance of the Piezo1 channel in mechanotransduction, particularly in periodontal ligament cells, and will provide a foundation for future orthodontic treatment methods.^129^

The identification of Piezo channels not only enriches our understanding of mechanotransduction mechanisms but also highlights the ubiquitous essence and multifunctionality of mechanosensitive channels in organisms.^91^ Although the specific role of Piezo channels in the DH process is not yet fully understood, research on Piezo channels and their relationship with DH remains a hot topic, particularly in the context of osteogenesis.^133^ Exploring how Piezo channels participate in and regulate the DH process presents a promising direction for future research.

### TRP channels

TRP channels constitute a family of non-selective cation channels, many of which are highly permeable to calcium ions.^134^^,^^135^ Under mechanical force stimulation, TRP channels open, allowing calcium ions to enter the cell, thereby increasing intracellular calcium concentration and regulating cellular functions and signal transduction^93^^,^^136^, ^137^, ^138^ (Fig. 5). The TRP channels were first discovered in the 1990s, with the TRPV1 channel identified by Caterina et al in 1997, opening the way to discovering other TRP channels involved in sensory processes.^139^ Based on the sequence similarity of TRP proteins, they can be divided into seven subfamilies of channels (TRPA, TRPC, TRPML, TRPM, TRPN, TRPP, TRPV), which are tetrameric cation channels.^140^ The TRPV (vanilloid) subfamily includes six different subtypes, TRPV1–6, closely associated with the mechanotransduction process.^141^ Current research primarily focuses on TRPV1 and TRPV4, especially TRPV4, due to its extensive expression in bone, cartilage, and synovial tissues.^136^^,^^141^ TRPV4 channels, expressed in both osteoblastic and osteoclastic cells, can regulate bone morphology and density, and mice lacking TRPV4 exhibit resistance to bone loss under non-load-bearing conditions.^142^ Previous studies highlighted the expression of TRPV4 in *Caenorhabditis elegans* PCA-type sensory neurons, emphasizing its role as a crucial mechanosensor involved in mating behavior.^143^ The role of TRPV4 as a critical mechanosensitive ion channel has gained attention in recent research.^144^ TRPV4 is involved in mediating oscillatory fluid shear-induced mechanotransduction in MSCs, particularly localizing to the primary cilium, which is essential for mechanical sensing.^145^ Study shows that TRPV4 is necessary for translating mechanical forces into calcium signaling, thereby promoting early osteogenic gene expression and contributing to MSC differentiation into osteoblasts, a process vital for skeletal homeostasis and bone regeneration.^146^ In MSCs with defective primary cilia, the osteogenic response is significantly impaired, emphasizing the importance of this structure in mechanotransduction.^145^ These findings provide promising therapeutic opportunities for targeting stem cell mechanotransduction in bone and musculoskeletal regeneration. Recent studies demonstrate that mechanosensitive osteocytes adapt to mechanical stimuli by regulating sclerostin, a bone formation inhibitor. A microtubule-dependent mechanotransduction pathway links fluid shear stress to reactive oxygen species (ROS) and calcium (Ca^2+^) signaling, reducing sclerostin levels.^147^^,^^148^ Microtubule stabilization through detyrosination influences osteocyte mechanosensitivity, while nicotinamide adenine dinucleotide phosphate (NADPH) oxidase 2 (NOX2) activation generates ROS that stimulates the TRPV4 calcium channel, leading to calcium influx and further activation of CaMKII.^147^^,^^148^ These insights reveal potential targets for enhancing osteocyte mechanotransduction and improving bone quality. Additionally, a study using mechanical tooth movement and a rat *in vitro* compression force model found that mechanical force regulated the characteristics of MSCs (periodontal ligament stem cells) during bone remodeling through TRPV4 activation of the extracellular signal-regulated kinase (ERK) pathway.^149^ Recent studies indicate that TRPV4 mutations are associated with a range of skeletal dysplasias and arthropathies.^150^ The absence of TRPV4 channels can lead to the development of osteoarthritis and a decline in osteocyte function.^151^, ^152^, ^153^, ^154^ These findings underscore the pivotal role of TRPV4 as a mechanosensitive ion channel in bone formation and metabolic regulation.^151^, ^152^, ^153^, ^154^

Additionally, transient receptor potential melastatin 7 (TRPM7) is increasingly recognized for its crucial role in mechanotransduction and the promotion of osteogenesis^155^ (Fig. 5). TRPM7 plays a critical role in MSCs' mechanotransduction by directly sensing membrane tension. Mechanical stimulation activates Ca^2+^ influx through TRPM7, which further triggers calcium release from the endoplasmic reticulum via the inositol trisphosphate receptor type 2 (IP3R2).^156^ This cascade leads to the translocation of NFATc1 into the nucleus, promoting the expression of osteogenic genes and driving bone formation. This pathway underscores the central role of TRPM7 in MSC mechanical stimulation-induced osteogenesis, highlighting its importance in bone regeneration.^156^ In addition, TRPM7 has been shown to mediate osteogenic differentiation in mesenchymal stromal cells under intermittent fluid shear stress through the TRPM7-osterix axis, highlighting its importance in mechanotransduction pathways that regulate mesenchymal stromal cell fate decisions.^155^ Recent preliminary findings have shown that the TRPM8 channel promotes osteogenic differentiation of human MSCs.^157^ This study suggests a potential role for TRPM8 in mechanotransduction-mediated osteogenesis, although current research on this topic is limited, and further investigations are needed.

In summary, TRP channels like TRPV4 and TRPM7 are key in mechanotransduction, converting mechanical signals into cellular responses that promote bone regeneration through calcium signaling and osteogenic pathways. Their roles in osteocyte function and BMSC differentiation make them critical targets for DH. Future research could explore the therapeutic potential of modulating TRP channels for enhancing bone healing and treating skeletal disorders, opening new avenues for interdisciplinary applications in regenerative medicine.

### ENaCs

The ENaC family represents a crucial group of sodium channels, which include not only vertebrate ENaCs and acid-sensitive channels (ASICs) but also DEGs in nematodes, pickpocket (PPK) in fruit flies, and peptide-gated sodium channels (HyNaCs) in hydra.^18^^,^^158^^,^^159^ Under mechanical force stimulation, ENaCs open, followed by entry of sodium ions to the cell, leading to membrane depolarization, changes in ion balance, and regulation of cellular functions and signal transduction.^93^^,^^160^ ENaC was discovered in the late 1980s and cloned by Canessa et al in 1994, significantly advancing the understanding of sodium balance and fluid homeostasis.^161^ These channels are assembled from α, β, γ, and δ, four homologous subunits, into a heterotrimeric structure, and their activity is regulated by proteolytic action and mechanical forces.^162^ A previous study demonstrated that ASICs responded to shear stress and acidic pH, manifesting as transient current increases, highlighting their potential as mechanical sensors.^163^ Other studies found ENaC activity regulated by calpain-2 proteolysis of the myristoylated alanine rich protein kinase C substrate (MARCKS) protein.^164^ In the regulation of bone formation, nitric oxide synthases, voltage-sensitive calcium channels, and cyclooxygenase-2 have been shown to be involved in the functioning of ENaCs.^160^ ENaCs may also play a role in regulating functional changes in osteoblastic cells driven by sodium under low osmotic pressure.^160^^,^^165^^,^^166^ In addition, in rat osteoblastic cells, 8-pCPT-cGMP through the cGMP/PKG II pathway regulates the expression of ENaCs, promoting the proliferation, differentiation, and expression of osteogenic genes in osteoblastic cells.^167^ Recent studies have shown that ENaCs play a significant role in diseases such as osteoporosis, skeletal muscle atrophy, and fractures.^160^

Overall, although ENaCs are one of the main mechanosensitive channels, there is little research on ENaCs in relation to osteogenesis or DH. Their role remains largely unknown and requires further exploration in future studies.

## Translating mechanical signals into histogenesis signals

### The extracellular matrix in signal transduction

The extracellular matrix (ECM) plays a pivotal role in converting mechanical signals into biochemical signals that promote bone growth, acting as a crucial physical medium between cells and their external environment.^168^^,^^169^ The ECM is composed of a network of proteins produced by cells, including major components like collagen, fibronectin, fibrin, and elastin, which form fibrous structures that endow it with unique mechanical properties.^169^, ^170^, ^171^ These assembled fibers allow cells to modify the mechanical signals they receive by converting soluble ECM proteins into insoluble fibers with distinct mechanical properties.^168^ Current research indicates that the ECM activates a series of intracellular signaling pathways that convert mechanical signals into internal biochemical signals, often involving cellular cytoskeletal actin, cell adhesion molecules, and extracellular signal-regulated kinases.^172^

Despite extensive studies on the ECM's role as a mediator of mechanical signal transduction, the mechanisms by which ECM sensitivity to mechanical signals is regulated remain unclear. Furthermore, the specific roles of different ECM components in mechanical signal transduction and how cells adjust the composition and function of ECM to adapt to various mechanical environments are to be elucidated.

### Mechanical signal transduction through the cell membrane

The cell membrane acts as a crucial interface that mediates the cell's response to external and internal mechanical stimuli and the subsequent biochemical signal transduction.^173^ Singer et al first proposed the fluid mosaic model, emphasizing the fluid nature of the cell membrane.^174^ This model was further refined later on, to emphasize that it consists primarily of a mosaic of phospholipids, cholesterol, and proteins, all highly fluid.^175^ This fluidity allows the cell membrane to adapt to changes in mechanical conditions, maintaining cellular morphology and mechanical performance.^173^ Simultaneously, this fluid nature is essential for the function of mechanosensitive channels, as it allows them to respond dynamically to mechanical stimuli. Mechanosensitive channels, such as ENaC, TRP, and Piezo, located on the cell membrane, form ion-conductive pathways that respond to changes in mechanical forces.^88^^,^^176^ The known eukaryotic mechanosensitive channels include ENaC, TRP, and Piezo,^18^ which play key roles in mechanical force transduction. These cell membrane channels recognize, convert, and transmit external mechanical signals, thereby regulating various physiological processes.^177^ In addition to mechanosensitive channels, other components of the cell membrane, such as integrins, fibronectins, and osteocalcin receptors, also participate in the transduction of mechanical signals.^168^

### Conduction of mechanical force signal pathways

**Figure 6:**
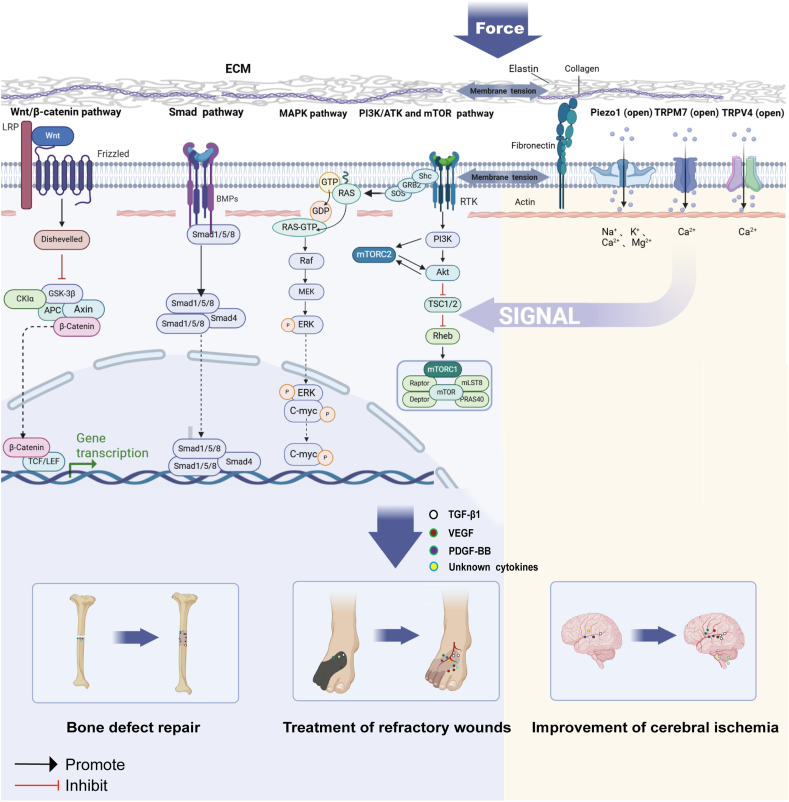
Mechanotransduction and signalings during distraction histogenesis. The mechanical force acts on the extracellular matrix and cell membranes, subsequently affecting mechanosensitive channels (such as Piezo1, TRPM7, TRPV4). These channels, upon receiving mechanical signals, activate corresponding signaling molecules. These molecules promote cell proliferation and differentiation by activating a series of complex signaling pathways (such as Wnt/β-catenin, Smad, MAPK, PI3K/AKT, and mTOR) and secrete growth factors like TGF-β1, PDGF-BB, and VEGF. Subsequently, these growth factors are transported through the peripheral blood to distant sites of injury, facilitating bone elongation or reconstruction, as well as the formation and repair of vascular and skin tissues. Piezo, piezoelectric element; TRP, transient receptor potential; MAPK, mitogen-activated protein kinase; PI3K, phosphatidylinositol 3*'* -kinase; AKT, protein kinase B; mTOR, mechanistic target of rapamycin; TGF-β1, transforming growth factor-beta 1; PDGF-BB, platelet-derived growth factor-BB; VEGF, vascular endothelial growth factor.

Cells' perception of and response to mechanical forces involve the activation and regulation of multiple signaling pathways, covering many molecular mechanisms from the cell membrane to the cytoplasm, and further, to the nucleus. Many of these mechanisms have been studied and involve multiple signaling pathways and molecular routes^178^ (Fig. 6). We summarize the key signaling pathways in DH in Table 4.

**Table 4:** Summary of key signaling pathways in distraction histogenesis.

Signaling pathway	Role in distraction histogenesis	Main components	References
Wnt/β-catenin	Promotes osteoblast differentiation, bone formation, and fracture healing.	WNT ligands (WNT4, WNT10A), receptors (FZD1, FZD2, LRP5, LRP6), β-catenin, DKK1, sFRP1, sFRP2,	181,182,185,186
Smad	Mediates TGF-β and BMP signaling, crucial for osteoblast proliferation and differentiation.	TGF-β, BMP-2, BMP-4, Smad2/3, Smad4	200,203,204,207,208
MAPK	Regulates cell proliferation, differentiation, migration, and apoptosis during bone regeneration	ERK, JNK, p38	213, 214, 215, 216
PI3K/AKT and mTOR	Controls cell growth, survival, metabolism, and angiogenesis, vital for bone regeneration.	PI3K, AKT, mTORC1, mTORC2	76,218,220,222
HIF-1	Enhances osteogenesis and angiogenesis under hypoxic conditions.	HIF-1	16,224, 225, 226
CXCR4/SDF-1	Recruit mesenchymal stem cells to promote bone and tissue regeneration.	CXCR4, SDF-1	227, 228, 229, 230
RhoA/ROCK-TAZ	Regulating the osteogenesis process.	RhoA, ROCK, TAZ	231
YAP/TAZ-Notch	Regulation of H-type endothelial cells to promote osteogenesis.	YAP, TAZ, Notch	232
PGE2-hypothalamic neuroendocrine	Regulating bone formation, integrating mechanical stimuli, and modulating metabolic processes.	PGE2, EP receptors, NPY, CREB, sympathetic nervous system	233, 234, 235

Note: MAPK, mitogen-activated protein kinase; PI3K, phosphatidylinositol 3' -kinase; AKT, protein kinase B; mTOR, mechanistic target of rapamycin; HIF-1, hypoxia inducible factor-1; CXCR4, chemokine C-X-C motif receptor 4; SDF-1, stromal cell-derived factor 1; RhoA, Ras homolog family member A; ROCK, Rho-associated kinase; TAZ, tafazzin; YAP, Yes-associated protein; PGE2, prostaglandin E2; EP, prostaglandin E2 receptor; ERK, extracellular signal-regulated kinase; JNK, c-Jun N-terminal kinase; TGF-β, transforming growth factor-beta; BMP: bone morphogenetic protein; FZD, Frizzled; LRP, low-density lipoprotein receptor-related protein; sFRP, secreted frizzled-related protein; DKK1, Dickkopf-related protein 1; NPY, neuropeptide Y; CREB, cAMP-response element binding protein.

#### Wnt/β-catenin signaling pathway

The Wnt/β-catenin signaling pathway is one of the most critical cellular biological pathways in the osteogenic mechanotransduction process. The earliest study of the role of the Wnt/β-catenin pathway in mechanotransduction was on mouse mammary tumor formation in the early 1980s.^179^^,^^180^ Subsequent research revealed that Wnt/β-catenin signaling plays a central regulatory role in establishing and remodeling bone morphology, particularly in bone tissue's response to mechanical loads.^181^, ^182^, ^183^ This pathway positively influences the conversion of MSCs to osteoblasts and their maturation, directly participating in callus remodeling and bone repair.^184^ Studies showed that induced expression of Wnt7b promoted bone formation in aged mice and enhances fracture healing.^182^^,^^185^ Kasaai et al, in 2012, first revealed the activation of the Wnt pathway during the DH process in mice.^186^ They found that WNT ligands (WNT4 and WNT10A), receptors (FZD1, FZD2, LRP5, and LRP6), β-catenin, and its antagonists (DKK1, CTBP1, CTBP2, sFRP1, sFRP2, and sFRP4) were up-regulated during DH and down-regulated during consolidation, providing clues that WNT could be a potential therapeutic target to accelerate bone regeneration.^186^ Further studies showed that the chitosan/si-CKIP-1 silencing technique could activate the Wnt3a/β-catenin signaling pathway in a rat mandibular DH model, thereby promoting bone formation.^187^

Additionally, another study verified the role of notoginsenoside in promoting endothelial progenitor cell angiogenesis and emphasized the importance of the Wnt/β-catenin pathway in resolving blood supply issues in DH, opening new research and clinical application directions in the field of bone regeneration.^188^ Furthermore, a previous study highlighted the significant role of the Wnt pathway in DH.^189^ By establishing a DH model in rats and using the Wnt pathway inhibitor recombinant rat Dickkopf-related protein 1 (rrDkk1), the authors observed an increased expression of Wnt pathway components during the DH process.^189^ However, the application of rrDkk1 inhibited these signaling components and limited the healing process, indicating that inhibiting the Wnt pathway should be avoided in DH treatment.^189^ Moreover, recent studies indicate that thrombin peptide 508 or epidermal growth factor-like domain-containing protein 6 (EGFL6) can promote bone regeneration in distraction osteogenesis by activating the Wnt/β-catenin signaling pathway.^67^^,^^190^, ^191^, ^192^ In addition, the large tumor suppressor 1 (LATS1)/YAP1 axis has also been found to control bone regeneration in distraction osteogenesis through activation of the Wnt/β-catenin pathway.^193^ These findings reveal novel molecular mechanisms, suggesting that specific growth factors and proteins play crucial roles in enhancing bone formation and tissue repair through modulation of the Wnt/β-catenin pathway. This provides potential targets and new therapeutic strategies for improving the efficacy of distraction osteogenesis therapy.

Similarly, in experiments on orthodontic tooth movement, the activation of the Wnt/β-catenin pathway was observed. Studies found that orthodontic forces can elevate the levels of Runx2 mRNA and β-catenin in osteoblastic cells.^194^ In a study of the DH rat model, the authors found that the gene expression of some components of the Wnt pathway changed during the DH process.^195^ These findings highlight the activating role of the Wnt pathway in DH and its potential as a target for accelerating bone regeneration.

#### Smad signaling pathway

The Smad protein family, discovered through genetic screening in invertebrates, consists of eight members, classified into three types: receptor-regulated Smads (R-Smads): Smad1, 2, 3, 5, and 8; the common mediator Smad (Co-Smad): Smad4; and inhibitory Smads (I-Smads): Smad6 and Smad7.^196^, ^197^, ^198^, ^199^ The receptor-regulated Smads are closely related to osteogenesis. Smad2 and Smad3 are specific to TGF-β and activin signals, whereas Smad1, Smad5, and Smad8 respond to BMP signals.^200^^,^^201^ The Smad protein family acts as intermediary molecules that transmit signals from the cytoplasm to the nucleus following the binding of TGF-β to its receptor, playing a significant role in signal transduction and regulating downstream target gene transcription.^202^^,^^203^

BMP is a subgroup of the TGF-β superfamily that plays a crucial role in bone healing and traction-induced osteogenesis.^204^ Research indicates that during DH, mechanical tensile stress induces the expression of BMP-2 and BMP-4, but does not trigger the expression of BMP-6, BMP-7, or growth differentiation factor 5 (GDF-5) mRNA.^205^ Radomisli et al established a femoral distraction model in rats, revealing that the early stages of distraction osteogenesis under mechanical load led to the elevated expression of type I and type II collagen, BMP-2/4, and osteocalcin.^206^ Then, Farhadieh and colleagues established a sheep mandibular distraction model and found high expression of BMP-2, BMP-4, and Smads.^207^ Subsequently, Haque et al established a rabbit tibial DH model and observed high expression of BMP signaling during the DH process.^208^ In addition, Khanal et al, using a rat mandibular bone DH model, found that the expression of BMP-2, -4, and Smads 1, 5, 8 increased significantly during distraction and gradually decreased during consolidation, suggesting that the Smad signaling pathway might play an important role in mandibular distraction.^200^ In two recent studies, high expression of BMP and Smads during DH was also observed.^203^^,^^204^ The above studies suggest that DH may promote osteoblast proliferation and participate in traction-induced osteogenesis by regulating the expression of BMP/Smad signaling molecules.

#### Mitogen-activated protein kinase signaling pathway

The MAPK pathway is a critical link between the cell surface and the nucleus. It regulates cell proliferation, differentiation, migration, and death, as well as bone formation.^209^, ^210^, ^211^ This pathway encompasses various cellular signaling pathways, including ERK, c-Jun N-terminal kinase (JNK), and p38. Studies compared the use of the p38 activator anisomycin and p38 inhibitors in a rat mandibular bone distraction model and found that anisomycin promoted new bone formation during DH, while p38 inhibitors reduced new bone formation.^212^ This result indicates that anisomycin promotes the recruitment of MSCs in the distraction gap, providing a new strategy for bone regeneration. Moreover, using *in vivo* and *in vitro* distraction models, they explored how static strain affects the migration of BMSCs and the role of the p38/matrix metalloproteinase-2 (MMP-2) axis in this process.^213^ They first demonstrated the importance of the p38/MMP-2 axis in regulating BMSC migration under static mechanical strain.^213^ Additionally, Tang et al found that Fibroblast growth factor 9 (Fgf9) negatively regulates osteoblast and osteoclast formation through MAPK and PI3K/AKT pathways, confirming the role of the MAPK pathway in osteogenesis.^214^ Further, research has shown that interleukin 17F (IL-17F) or ZINC40099027 mediates osteoblast proliferation, differentiation, and mineralization through the MAPK/ERK1/2 pathway, a critical step in bone regeneration, suggesting that IL-17F or ZINC40099027 may be a potential target for treating bone loss diseases.^107^^,^^215^ Additionally, in orthodontic treatment, periodontal tissue remodeling is closely related to the activation of the ERK1/2 and p38 MAPK signaling pathways and the up-regulation of bone-related genes, providing a deeper understanding of the mechanisms of periodontal tissue changes during orthodontic treatment.^216^ Moreover, a study involving cyclic tensile stress applied to rat calvarial osteoblasts revealed that ERK1/2 and signal transducer and activator of transcription 3 (STAT3) are sequentially activated by tensile loading, both contributing to osteogenesis during this process.^72^ These findings underline the multifaceted role of the MAPK signaling pathway in DH, providing a theoretical basis for future use of this pathway to promote bone regeneration and treat related diseases.

#### PI3K/AKT and mTOR signaling pathways

In DH, the interaction between the mechanistic target of rapamycin (mTOR) and PI3K/AKT signaling pathways has significant biological significance, as these pathways jointly regulate key processes in bone regeneration.^182^^,^^217^ mTOR is a special serine/threonine protein kinase belonging to the family of PI3K-related kinases, forming two different complexes: mTOR complex 1 (mTORC1) and mTOR complex 2 (mTORC2).^182^^,^^217^ AKT is not only a component of the PI3K pathway but also closely associated with the activation of mTORC1.^218^ AKT can directly or indirectly activate mTORC1 by inhibiting tuberous sclerosis complex 1/2 (TSC1/2), while on the other hand, mTORC2 is also involved in the phosphorylation and activation of AKT, indicating the role of mTORC2 in the PI3K/AKT pathway.^218^

The mTOR signaling pathway plays a key role in cell division and the differentiation of MSCs. Animal models of DH have shown a significant increase in the expression of the mTOR gene in the new bone formation area, confirming the activation of the mTOR signaling pathway in DH.^219^ Wang et al demonstrated how mechanical stretching promoted the expression of osteogenic differentiation markers in MG-63 osteoblast-like cells, accompanied by increased phosphorylation of mTOR and nuclear factor kappa-B (NF-κB) p65 and their migration to the nucleus.^220^ This study first revealed the interaction between mTOR and NF-κB under mechanical stretching, indicating their key roles in regulating the cellular homeostasis of osteoblasts under mechanical stretch.

The PI3K/AKT pathway, involved in regulating various biological processes including cell growth, survival, metabolism, and angiogenesis, plays a central role in cellular functions through the activation of AKT.^221^ Angiogenesis is critical for the success of DH, where endothelial progenitor cells play a vital role. Research shows that under DH and hypoxic conditions, the angiogenic capacity of endothelial progenitor cells increases with the elevation of m^6^A methylation and methyltransferase-like 3 (METTL3) levels, while METTL3 enhances endothelial progenitor cell angiogenesis by activating the PI3K/AKT pathway, thus, promoting bone regeneration.^222^ Additionally, using a canine DH and bone defect model, it was found that the expression of cluster of differentiation 34 (CD34), CD133, HIF-1α, and heat shock protein 20 (Hsp20) at the mandibular distraction osteogenesis site was up-regulated, revealing a new mechanism by which Hsp20 regulates endothelial progenitor cells in a hypoxic environment through AKT activation.^223^ Further, canine endothelial colony-forming cell-derived exosome thrombospondin 1 (THBS1) mediated angiogenesis and osteogenesis in DH through the PI3K/AKT/ERK pathway.^76^ These findings highlight the importance of the PI3K/AKT pathway in promoting bone regeneration and angiogenesis during DH, providing new research directions and treatment strategies to accelerate bone regeneration. These results underline the synergistic action of mTOR and PI3K/AKT in DH. They not only individually affect bone cell growth and differentiation but also strengthen the response to mechanical and biochemical signals through their interaction. Therefore, the interaction between mTOR and PI3K/AKT in DH provides a new perspective for understanding the molecular mechanisms of bone regeneration and may guide future clinical treatment strategies, especially in promoting bone repair and regeneration.

#### Other signaling pathways

In addition to the main pathways discussed, other pathways, such as the HIF-1α pathway, have also shown their ability to promote bone formation in DH.^16^^,^^224^, ^225^, ^226^ Furthermore, the chemokine C-X-C motif receptor 4 (CXCR4)/stromal cell-derived factor 1 (SDF-1) pathway also deserves attention.^227^^,^^228^ In a rat mandibular DH model, recruitment of exogenous MSCs was mediated through the SDF-1/CXCR4 pathway.^229^ Similarly, tibial transverse transport surgery facilitated the healing of diabetic foot ulcers through the activation of the SDF-1/CXCR4 signaling pathway.^230^ In addition, a recent study has shown that the RhoA/Rho-associated kinase (ROCK)-tafazzin (TAZ) axis can regulate bone formation within the cranial suture osteogenesis.^231^ Another study indicates that tensile stress activates and exosomal transfer of the YAP/TAZ-Notch circuit designates H-type endothelial cells for segmental bone regeneration.^232^ These findings warrant future studies to delve deeper into the molecular mechanisms of these signaling pathways.

Recently, the signals of osteocytes responding to mechanical stress have been preliminarily revealed to be processed and interpreted in the brain. Lv et al demonstrated how mechanotransduction through skeletal interoception regulated bone and fat metabolism via the prostaglandin E2 (PGE2)/prostaglandin E2 receptor 4 (EP4)-hypothalamic pathway. This shows how mechanical signals influence systemic energy balance, linking DH with metabolic control.^233^ Guo et al further revealed that unloading-induced skeletal interoception reduced PGE2 levels, leading to bone loss through hypothalamic neuropeptide Y (NPY) signaling.^234^ Their subsequent research confirmed that mechanical stress-induced PGE2 activates hypothalamic cAMP-response element binding protein (CREB), promoting bone formation and regulating sympathetic activity.^235^ This study underscores the critical role of mechanotransduction in DH and its implications for conditions such as osteoarthritis. These studies collectively illustrate the vital role of mechanotransduction in musculoskeletal regeneration and its broader systemic effects through neuroendocrine pathways.

### Perspective

Current research on bone disease, DH, and mechanosensitive channels show how mechanical signals are transmitted through the extracellular matrix and cell membrane, activating a variety of signaling pathways, including Wnt/β-catenin, mTOR, MAPK, Smad, and PI3K/AKT. These signaling pathways play significant roles in regulating bone cell proliferation, differentiation, and matrix synthesis. However, challenges and gaps still exist. For example, research on how mechanosensitive channels is activated during DH is still scarce, and a more in-depth understanding of the roles of the downstream signaling pathways activated by mechanosensitive channels in bone growth and tissue regeneration, as well as their interactions and regulatory mechanisms, is still needed. Additionally, current research on DH primarily focuses on *in vitro* experiments and small animal models, with large-scale clinical studies being rare. Therefore, more clinical research is needed to verify its applications in bone and tissue regeneration.

With a deeper understanding of mechanotransduction mechanisms, we anticipate that DH will be further optimized and expanded in its applications in treating orthopedic diseases, craniofacial disorders, ischemic diseases of the lower limbs, and refractory ulcers, among other areas. Moreover, considering the potential of DH in the regeneration of blood vessels, skin, and other tissues, interdisciplinary collaboration will be key to driving innovation and applications of DH. With new research directions being explored, DH is poised to become a powerful tool in promoting tissue regeneration across various fields.

## CRediT authorship contribution statement

**Xiajie Huang:** Writing – review & editing, Writing – original draft, Validation, Resources, Data curation. **Wenjun Hao:** Writing – review & editing, Writing – original draft, Data curation. **Yangzhou Mo:** Validation, Software, Formal analysis. **Xinyun Liang:** Validation, Formal analysis, Data curation. **Xiaomei Wu:** Formal analysis, Data curation. **Daofu Zeng:** Investigation, Data curation. **Yubin Mo:** Data curation. **William Lu:** Writing – review & editing. **Di Chen:** Writing – review & editing, Conceptualization. **Yan Chen:** Writing – review & editing, Writing – original draft, Supervision, Conceptualization.

## Funding

This study was supported by grants from the National Natural Science Foundation of China (No. 82360429 and 82060406), Natural Science Foundation of Guangxi, China (No. 2023GXNSFAA026474), Advanced Innovation Teams and Xinghu Scholars Program of Guangxi Medical University, China Postdoctoral Science Foundation (No. 2019M650235), and Key R&D Project of Qingxiu District, Nanning, Guangxi, China (No. 2021003).

## Conflict of interests

The authors declared no conflict of interests.
